# Review of measurements and imaging of cytochrome-c-oxidase in humans using near-infrared spectroscopy: an update

**DOI:** 10.1364/BOE.501915

**Published:** 2023-12-14

**Authors:** Georgina Leadley, Topun Austin, Gemma Bale

**Affiliations:** 1 Department of Paediatrics, University of Cambridge, UK; 2 Department of Engineering, University of Cambridge, UK; 3 Department of Medical Physics and Biomedical Engineering, UCL, UK; 4 Department of Physics, University of Cambridge, UK

## Abstract

This review examines advancements in the measurement and imaging of oxidized cytochrome-c-oxidase (oxCCO) using near-infrared spectroscopy (NIRS) in humans since 2016. A total of 34 published papers were identified, with a focus on both adult and neonate populations. The NIRS-derived oxCCO signal has been demonstrated to correlate with physiological parameters and hemodynamics. New instrumentation, such as systems that allow the imaging of changes of oxCCO with diffuse optical tomography or combine the oxCCO measurement with diffuse correlation spectroscopy measures of blood flow, have advanced the field in the past decade. However, variability in its response across different populations and paradigms and lack of standardization limit its potential as a reliable and valuable indicator of brain health. Future studies should address these issues to fulfill the vision of oxCCO as a clinical biomarker.

## 1. Introduction

A robust non-invasive measurement of oxidized cytochrome-c-oxidase (oxCCO) has long been known to have potential to provide direct insight into brain health since its first description by Jöbsis in 1977 [1]. Near-infrared spectroscopy (NIRS) is capable of measuring changes in the concentration of several biological markers (chromophores) instantaneously and non-invasively. A review published by Bale et al. in 2016 [2] summarized the state of the field of measuring oxCCO in humans using NIRS. This review provides a follow-up to that paper, highlighting the progress made within the last 7 years. In the years since the 2016 review, Jöbsis’ paper has been cited an additional 2,000 times for a total of 5,000 citations.

The utility of NIRS lies in its ability to take advantage of the relative transparency of biological tissue in the near-infrared range (600-900nm) and the different absorption spectra of key chromophores whose absorption varies with oxygenation status. The two principal chromophores are oxy- and deoxy-hemoglobin (HbO and HbR) which provide information on the oxygen supply and consumption from the vasculature. A third chromophore, oxCCO is found in the mitochondria of most cells (although not the red blood cells which contain HbO and HbR) and thus provide a measure of cellular metabolism. Simultaneous measurements of oxCCO and hemoglobin signals have the potential to provide information on oxygenation, metabolism and hemodynamics and the relationship between each of these parameters.

NIRS, and principally the measurement of changes in HbO and HbR and the derived measure of cerebral tissue oxygenation, StO_2_ has been widely used in research and clinical practice [3,4]. By using multiple light sources and detectors it is possible to assess changes in regional hemodynamics resulting from brain activity at rest or in response to stimuli (functional NIRS – fNIRS). While numerous published works report only on changes in concentration of the oxygenation status of hemoglobin [5–9], this review will focus solely on developments and studies that include a measurement of oxCCO.

Compared to NIRS studies measuring HbO and HbR, there are relatively fewer studies including oxCCO. The absorption characteristics of oxCCO and the fact that oxCCO is less abundant by an order of magnitude in tissue make it difficult to measure oxCCO alongside HbO and HbR with conventional 2 or 3 wavelength systems. However, technologies utilizing multi-wavelength or broadband technologies have provided new insights into oxCCO physiology by being able to resolve the concentration of oxCCO and separate it out from the HbO/HbR signal.

Although the oxCCO signal is more difficult to resolve than HbO/HbR, measuring oxCCO confers some important advantages over HbO/HbR measurements alone. Signals from oxCCO are much less likely to suffer from scalp or skull contamination compared to those from hemoglobin, as CCO concentration within the brain is much higher than its concentration in extracerebral tissue [10]. This is particularly beneficial for functional NIRS (fNIRS) measurements which offer insight into brain activity and cortical functional connectivity. As a cellular chromophore it also offers direct assessment of cell function and health [11]. Many studies measuring oxCCO have focused on cellular oxygenation stress following acute hypoxia-ischemia in both neonates and adults [12–14]. Published works focusing on clinical NIRS measurements of oxCCO show that important pathophysiological data can be obtained from different patient populations, including sick newborn infants, people with epilepsy, dementia, and those undergoing cardiac surgery [15–18]. Using NIRS to understand the mechanistic basis for brain injury for example in neonates could aid in the development of more targeted novel neuroprotective strategies [19–21]. Another emerging use of fNIRS is in brain-computer interfaces (BCIs), which allow individuals to control external devices by directly communicating with computers using brain activity [22]. The oxCCO signal could provide an additional marker of neural activity for input into BCIs.

**Fig. 1.**
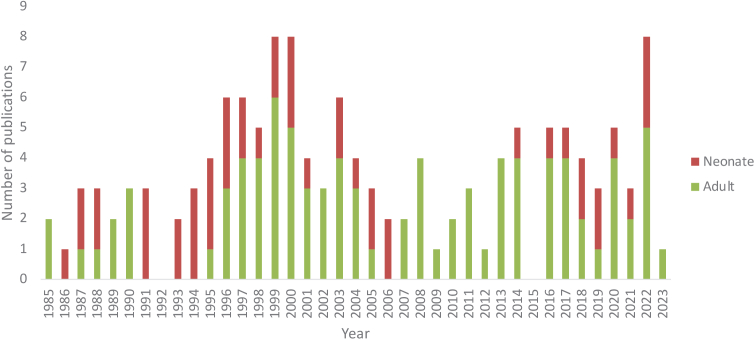
Bar chart displaying the number of published papers measuring oxCCO using NIRS from 1985–2022. Publications covered in the Bale et al. [2] review are greyed out.

The 2016 review by Bale et al. [2] reported the number of papers published using NIRS to measure oxCCO from 1977 through to 2014. Since its publication, a further 34 papers have been published measuring oxCCO in humans using NIRS, from 9 groups using 15 systems. Figure 1 shows the number of papers measuring oxCCO with NIRS in humans – adults and neonates – from 1985 to 2022. Data from 1985-2014 is taken from the review by Bale et al. [2]. There were no papers measuring oxCCO in humans using NIRS published is 2015.

There has been a continued effort and maintenance of an active research field shown in the number of studies measuring oxCCO in humans since 2016, partly due to the emergence of new research groups into this area (based primarily at Texas University, Western University Ontario, ETH Zurich, UZ Ghent and University of Michigan). Although there was a paucity of neonatal studies published between 2007-2015, there has been recent renewed interest, with 9 papers measuring oxCCO in neonates published in the past 4 years.

The aims of this review paper are summarized in a set of questions determined before commencement of the literature search:

• What advancements have been made in biomedical optics towards measurement or imaging of cytochrome-c-oxidase using NIRS?
• Are the observed trends in measured cytochrome-c-oxidase signals consistent across different studies?
• What do the papers measuring cytochrome-c-oxidase using NIRS reveal concerning trends and correlations between cytochrome-c-oxidase measurements and other measurements of systemic physiology?

This review focuses on oxCCO measurements reported in humans since 2016 using NIRS, with a view to whether these measurements have the capacity to contribute to a direct measurement of brain health and whether they have shown to be reliable across different studies. Measurements of oxCCO in muscles measured during cuff occlusions were also considered as they contribute to the understanding of underlying systemic physiology.

The search for relevant literature was conducted using several online scientific databases (WebMD, Medline, Scopus, Google Scholar, ResearchGate) using the key terms ‘NIRS’, ‘near-infrared spectroscopy’, ‘DOT’, ‘cytochrome-c-oxidase’, ‘CCO’, ‘aa3’ and ‘optics’. To decrease the number of papers returned, the term ‘photobiomodulation’ was excluded from the search criteria. The inclusion criteria were measurements of cytochrome-c-oxidase in either volunteer studies or patients of any kind and of any age. Exclusion criteria were NIRS measurements not including cytochrome-c-oxidase or measurements of cytochrome-c-oxidase in animals, as the animal oxCCO response will not be considered in assessment of the feasibility of the measurement for future clinical applications. This review conforms to PRISMA guidelines and was registered in PROSPERO (CRD42023421355).

The main parameters recorded for each paper in this review were:

• Population age group
• Population status (healthy/sick)
• Number of participants
• Wavelengths used to resolve cytochrome-c-oxidase changes
• Whether the system used is capable of imaging
• Type of paradigm used in study population to elicit/observe cytochrome-c-oxidase changes
• Nature of the cytochrome-c-oxidase response.

A total of 52 papers were initially screened. 17 papers were rejected due to either being included in the previous review by Bale et al. [2] or based on measurements in animals or phantoms. Appropriate papers were quality assessed for aspects such as methods, study population and author-declared conflict of interest. All papers that fit these criteria are included in the review.

## 2. Cytochrome-c-oxidase: a metabolic marker

**Fig. 2.**
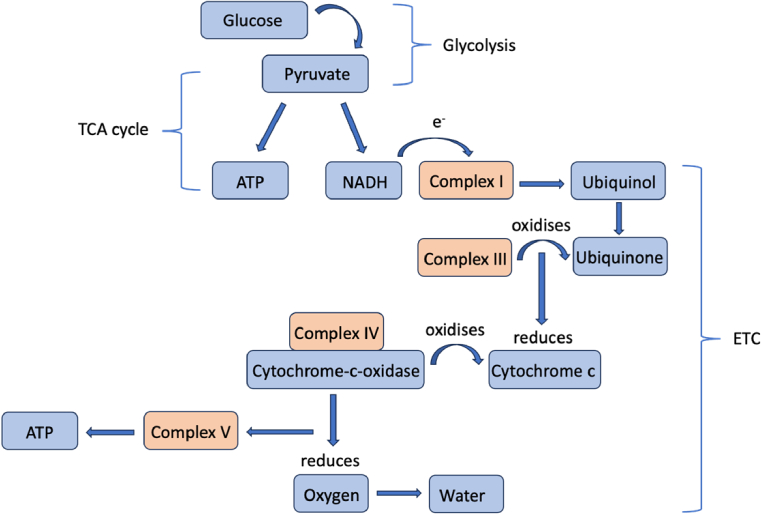
Diagram of electron transport chain processes indicating the method via which the redox potential drop across complexes I, III and IV drives ATP synthesis

Cytochrome-c-oxidase (CCO) is the last enzyme in the respiratory electron transport chain and found in the mitochondria of cells. CCO is key to the production of adenosine triphosphate (ATP) within cells and thus provides a direct measure of tissue metabolism [23]. During aerobic metabolism in cells, glycose is converted into pyruvate by glycolysis. Pyruvate then crosses into the mitochondria where it enters the tricarboxylic acid (TCA) cycle. As part of this process reduced nicotinamide adenine dinucleotide (NADH) is produced, which is an electron donor. At the mitochondrial membrane electrons are essentially ‘passed down’ an energy gradient in a series of redox reactions which are coupled to the transfer of protons (H+) across the membrane. The electron transfer chain (ETC) refers to a series of complexes embedded in the mitochondrial membrane facilitating these reactions. CCO is an enzyme and the terminal electron acceptor (complex IV) in the ETC where electrons are transferred to oxygen and protons to form water. The movement back of H + across the membrane at this point is coupled to the phosphorylation of adenosine diphosphate (ADP) to form ATP, which acts as an energy store to drive cellular processes in the body [24]. This whole process is called oxidative phosphorylation. The electron transport chain processes are detailed in Fig. 2.

Cytochromes are proteins characterized by a heme molecule and central iron atom where electron transfer occurs. The International Union of Biochemistry and Molecular Biology (IUBM) recognizes four types (a-d) which are represented across complexes I-V. Cytochrome c which is reduced by complex III, in turn donates its electron to CCO. The active sites of CCO are two copper a molecules (a and a3) and a copper dimer. Although all cytochromes have unique absorption spectra, it is the change in oxidation state of CCO that is used for in vivo measurements of cellular oxygenation with NIRS because of its spectral features in the near-infrared (NIR). A more in-depth review on cellular metabolism and the role of CCO in adenosine triphosphate (ATP) production and oxygenation can be found in the review by Bale et al. [2].

## 3. Methods for measuring cytochrome-c-oxidase with NIRS

The basic principle of NIRS is that light from an optical source is incident on the scalp, penetrating through the skull and extracerebral tissues and into the brain. This incident light is predominantly scattered by the tissues within the head with some absorbed by the chromophores resulting in a small proportion of light that can be detected by optical sensors located at the scalp. The modified Beer-Lambert Law [25] is used to convert the changes in measured light intensity (ΔA) to the changes in concentrations ([Δx]) of each chromophore of interest using the relevant extinction coefficient (ε):

$$\left[\begin{array}{c} \Delta [\text{HbO}]\\ \Delta [\text{HbR}]\\ \Delta [\text{oxCCO}] \end{array}\right]=\frac{1}{\text{pathlength}}{\left[\begin{array}{ccc} \varepsilon_{\text{HbO}}(\lambda_{1}) & \varepsilon_{\text{HbR}}(\lambda_{1}) & \varepsilon_{\text{oxCCO}}(\lambda_{1})\\ \varepsilon_{\text{HbO}}(\lambda_{2}) & \varepsilon_{\text{HbR}}(\lambda_{2}) & \varepsilon_{\text{oxCCO}}(\lambda_{2})\\ \vdots & \vdots & \vdots \\ \varepsilon_{\text{HbO}}(\lambda_{n}) & \varepsilon_{\text{HbR}}(\lambda_{n}) & \varepsilon_{\text{oxCCO}}(\lambda_{n}) \end{array}\right]}^{-1}\;\left[\begin{array}{c} \Delta A(\lambda_{1})\\ \Delta A(\lambda_{2})\\ \vdots \\ \Delta A(\lambda_{n}) \end{array}\right]$$

**Fig. 3.**
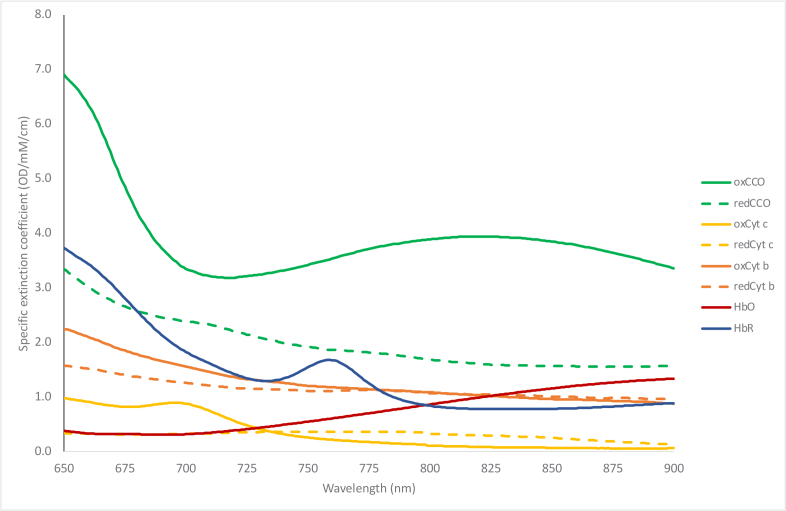
Specific extinction spectra of different cytochrome types, HbO and HbR [27].

As can be deduced from the equation, the number of wavelengths used to collect intensity measurements dictates the size of the extinction coefficient matrix and thus limits the number of chromophores that can be quantified. Additionally, assuming the system uses more than three wavelengths making the extinction coefficient matrix non-square, a pseudo-inverse is solved in the third term. Thus, utilizing a greater number of wavelengths will decrease the errors associated with the matrix pseudo-inversion and improve the accuracy of the resolved concentrations. Jöbsis observed that in its oxidized state (oxCCO), the copper dimer in the mitochondrial subunit IV enzyme shows an absorption band at 835nm, and in the absence of oxygen (during anoxia), CCO becomes fully reduced and this band disappears [1]. The extinction spectra of the different cytochromes found in tissue have been quantified [26], and the currently accepted values are reproduced in Fig. 3 [27]. CCO is 10 times less abundant than hemoglobin in the brain, so while in theory only three wavelengths would be required to calculate its concentration alongside the hemoglobin signals mathematically, in practice a greater number of wavelengths is required to ensure its accuracy.

Unlike the hemoglobins (the concentration of which is dependent on the hemoglobin concentration in the blood as well as blood flow to the brain), the total concentration of CCO is relatively constant within brain tissue, therefore only the difference spectrum is needed to quantify concentration changes in its redox state. It can be seen in Fig. 3 that CCO presents a unique opportunity for measuring the difference between its oxidised and reduced state (oxCCO-redCCO), as these extinction coefficients are substantially different from each other. Therefore, with the inclusion of additional wavelengths, a measurement of oxCCO can be obtained using the difference spectrum. Many NIRS systems used to obtain oxCCO measurements have utilized a broadband light source and have resolved chromophore concentrations over a large spectrum of the infrared range. The optimal wavelengths to use to reconstruct changes in oxCCO is still under debate, however, preclinical studies have showed that limiting broadband measurements from 780 to 900 nm improves the resolution of oxCCO as exclusion of shorter wavelengths lessens the contribution from other cytochrome types in the in-vivo measured spectrum commonly used (Fig. 3) [28].

## 4. Instrumentation

NIRS instrumentation used to measure oxCCO can be divided into two broad categories: broadband and discrete wavelength devices. A total of 15 systems were described across the 34 papers identified for this review. 6 of these utilized discrete wavelengths and 9 use broadband systems. Of the 6 discrete wavelength systems, 2 used time-resolved technology and 4 were fibreless.

Broadband systems typically use a white light source and detect the output intensity using fiber optic cables across a large section of the NIR window using a spectrometer. The number of measurement channels (source-detector pairs) utilized by NIRS systems is limited by cost and size of the spectrometers used. Across the papers found for this review, the maximum number of channels used was 32, which enabled image reconstruction of oxCCO using diffuse optical tomography (DOT) [29].

Using a small number of discrete wavelengths allows for a smaller, wearable system but potentially at the cost of a smaller signal to noise ratio (SNR). By frequency multiplexing different discrete wavelength sources (typically laser diodes or light emitting diodes (LEDs)), the requirement for a spectrometer is removed and replaced with photodiodes. There is a recent trend in NIRS towards wearable technology [30] and discrete wavelength systems which could allow for wearable oxCCO measurements. System acquisition time must also be considered for in-vivo measurements in order to obtain accurate information on the hemodynamic response to different tasks. The exact delay in response of HbO and HbR in healthy adults as measured using MRI range from 3-5s. The shape of the response is also known to vary with age, the cortical region measured, and whether the participant is awake or asleep [31].

**Fig. 4.**
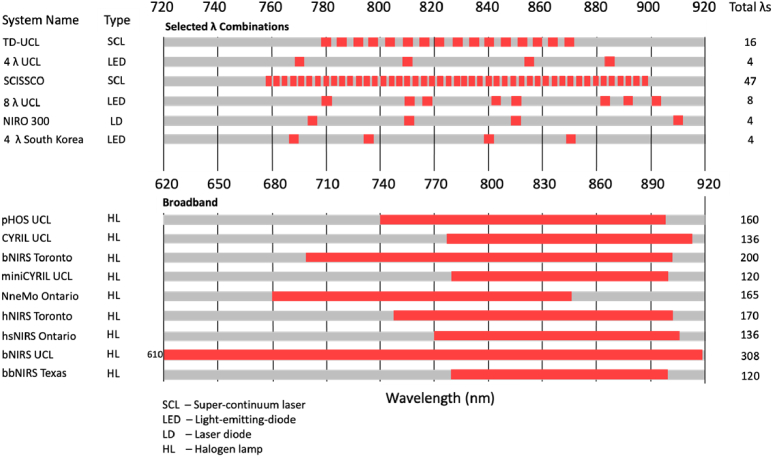
Chart visualising the number of systems used across the papers included in this review, including their source type, wavelengths and total number of wavelengths used to resolve oxCCO.

Assessment of the wavelengths used by different systems is critical when interpreting the nature and accuracy of NIRS measurements. The absorption of light in tissue is wavelength-dependent and can be used as an indicator of which wavelengths penetrate the furthest in tissue. As tissue comprises several different layers, the overall penetration depth of NIR light varies according to the absorption properties and thickness of each layer. Historically NIRS measurements of oxCCO have been made between 780-900nm [32]. Below 650nm, the pathlength of photons in tissue is limited due to high scattering and absorption effects and the extinction coefficients of oxCCO are very small compared to other absorbers in tissue [33]. Above 920nm, the absorption coefficient of water increases and prevents transmission of light through tissue. One 2015 study used a genetic algorithm to determine the minimum number of wavelengths required for a system to accurately resolve changes in oxCCO, with results suggesting a combination which evenly spans the 780-900nm spectral range [32]. A summary of the systems used in the studies found for this review is given in Fig. 4.

Diffuse optical tomography (DOT) is one such technique which has typically exploited laser diodes or light emitting diodes (LEDs) which use smaller electronics and detection hardware, resulting in a lighter and more portable system than broadband. There is a recent trend in NIRS towards wearable technology, which could present an opportunity for using DOT to image oxCCO in a broader range of functional tasks. There is currently a paucity of DOT systems capable of measuring oxCCO. Despite being vulnerable to movement artefacts, measurements of brain oxygenation with DOT are less sensitive to physiological artefacts than NIRS due to its depth sensitivity [34].

### 4.1. Continuous wave systems using optical fibers

**Fig. 5.**
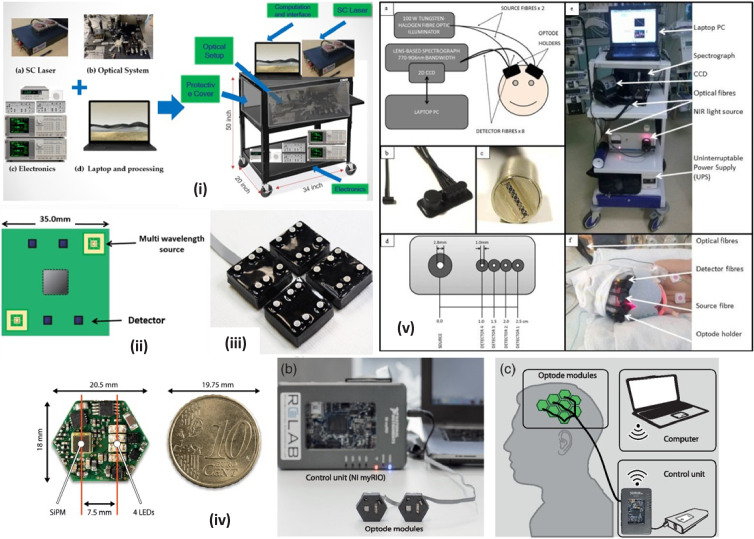
Diagram of different systems used in studies included in this review. **(i): SCISSCO.** High-level system schematic of cart-based SCISSCO prototype [41] , **(ii): 8λ UCL.** Schematic of single module containing two multi-wavelength sources and four detectors [42], **(iii): 8λ UCL.** photograph of the four-module system [42], **(iv): 4λ Zurich.** Visualization of the developed fNIRS instrument. (a) Close-up of the PCB next to a 10-cent euro icon. (b) Picture of the fNIRS instrument with two custom-made optode modules in rapid prototyped casings (front), the control unit, and a PC. (c) Conceptual sketch illustrating the arrangement of the system with eight modules placed over the motor cortex of an adult human. Optode modules are connected to a control unit (NI myRIO and a battery) allowing for wireless communication with a laptop computer [45] . **(v): CYRIL.** a) Instrumentation diagram with experimental set up. b) Detector optode with optode holder. c) Ferrule of detector fibres for input into spectrograph vertically. d) Optode holder design with dimensions of fibre diameters (all detector fibres have the same diameter) and source-detector distances. e) Image of CYRIL system in NICU. f) Image of CYRIL optodes on a subject [14] .

Continuous wave systems measure the change in light intensity through tissue and, by assuming (or estimating) a pathlength, determine the change in concentration of select chromophores. The use of optical fibers as light detectors allows coupling to a spectrometer, which enables recording high resolution spectra but limits the number of measurement channels due to size and cost. A summary of the systems included in this review is below:

• The UCL pHOS [35] comprises two components – a multi-distance broadband spectrometer and a multi-distance frequency domain spectrometer. A charge-coupled device (CCD) camera detects light at four separations (20, 25, 30 & 35mm) and resolves chromophore concentrations from the 35mm separation. The frequency domain component of the system estimates the absorption and scattering coefficients and hence differential pathlength of the tissue using four wavelengths.
• CYRIL (CYtochrome Research Instrument and appLication) is a lens-based 8-channel bNIRS system with two modules each comprising one source and 4 detectors (Fig. 5(v)). Broadband light is emitted by a tungsten halogen bulb and collected by a spectrometer within the 771-906nm range at a sampling rate of 1Hz. This system has been used in a wide variety of research papers and was one of the first portable broadband systems capable of measuring oxCCO in a clinical setting, first described in 2014 [14].
• miniCYRIL [36] is a miniature version of CYRIL, designed to be more portable so it can measure a wider variety of tasks in different settings. It comprises a miniature white light source, a customized miniature spectrometer, fiber-optic cables and a laptop for real-time visualization of results. However, the miniaturization results in the system only having one channel, limiting its usefulness for tasks involving both brain hemispheres.
• The bNIRS UCL system [37] has very similar hardware to CYRIL but is capable of measuring chromophore concentrations over a larger wavelength range (610-918nm). This system utilizes 16 channels with a sampling frequency of 2Hz, making it capable of measuring a greater array of brain locations at a faster rate than CYRIL.
• The bNIRS Toronto system [38] comprises a halogen light source delivering light via two source fibers, and two detector fibers transmitting measured light to two separate spectrometers. Light is sampled at 1Hz and used to resolve chromophore concentrations between 700-900nm with 30mm S/D separation. Efforts were made to improve the throughput of the spectrometer as modelled after a paper by Diop et al. [39] such as removing the input slit and reshaping the fiber bundles.
• The bbNIRS Texas system also offers two measurements channels and operates between 780-900nm with a consistent channel separation of 3cm.

### 4.2. Time resolved systems using optical fibers

Time resolved NIRS measures the time-of-flight of photons travelling through the tissue to quantify both absorption and scattering coefficients of the tissue. Time resolved NIRS offers several advantages over other techniques, such as eliminating errors associated with changes in scattering during measurements, the ability to accurately quantify the pathlength, and time gating the signal to improve depth sensitivity. The 2 time resolved systems are summarized below:

• The MAESTROS (TD-UCL) system [40] used 16 wavelengths to resolve oxCCO and were selected at 6nm intervals between 780-870nm. Light was transmitted to a single time-correlated single photon counting (TCSPC) card to measure the arrival time of the photons from 4 photomultiplier tubes at 40mm separation. The system had a sampling frequency of 0.5Hz. The use of a stable, high-intensity supercontinuum laser gives this system the advantage of a very high SNR and lower measurement errors, however the use of a single source and channel separation restricts the ability for depth measurements.
• The SCISSCO device [41] consists of a supercontinuum laser emitting between 759-897nm, and data is taken at 3nm intervals with a spectrometer to resolve HbO, HbR and oxCCO at a single 30mm separation. Due to the speed of the mechanical rotation of the grating in the spectrometer, data acquisition was limited to 17 seconds giving a sampling frequency of 0.06Hz. A schematic of the SCISSCO instrument is shown in Fig. 5(i), first constructed in 2022.

### 4.3. Fibreless systems

Fibreless systems use photodiode detectors to collect light intensity measurements at the tissue, as opposed to transmitting the light to a benchtop detector. The fibreless systems covered in this review are all continuous wave systems but have the advantage of being smaller, lower-cost and potentially more high-density than fiber-based systems. 4 fibreless systems were reviewed, 3 with 4 wavelengths and 1 with 8 wavelengths:

• The 8λ UCL system [42] comprises four square-shaped modules, each housing two multi-wavelength LED sources and four detectors. Each LED emits at 780, 811, 818, 842, 850, 882, 891 and 901 nm, based after the combinations found to be the most effective for resolving the oxCCO signal compared to a broadband spectrum [32]. A schematic and photo of the modules is shown in Fig. 5(ii & iii).
• The NIRO-300 [43,44], designed by Hamamatsu Photonics, delivers light at four distinct wavelengths (775, 810, 850 & 910nm) using pulsed laser-diodes. Three detectors are placed at different distances away from the source and data is acquired with a sampling rate of 1Hz. Concentration changes are measured using the modified Beer-Lambert Law with intensity data from one detector.
• An array of hexagonal imaging modules each housing one 4-wavelength LED and one detector are used in 4λ Zurich system [45]. The modules are made from flexible PCB and can be arranged to allow short (7.5mm) and long (20mm) separation channels. Each LED emits at 770, 810, 855 & 885nm (full width at half maximum (FWHM) 35-51nm) and are time-multiplexed in sequence, with each wavelength having an illumination time of 1.2ms. This combination of wavelengths was modelled after the optimal combinations found by Arifler et al [32] using a genetic hierarchy algorithm. A visualization of the system is provided in Fig. 5(iv).
• The 4λ system from South Korea [46] uses two four-wavelength sources and four photodiode detectors. Each source LED emits at 766, 796, 840 & 871nm (FWHM 27-50nm) and are also frequency multiplexed to allow for simultaneous illumination and therefore greater data acquisition rate (16Hz). The separation of all four channels was 3cm.

Since 2016, there has been an increase in the number of fibreless systems presented to measure oxCCO. Improvements in technology making detectors and circuit components smaller and cheaper has led to the ability to fit multiple sources and detectors in close proximity and has improved their measurement accuracy. Further modelling and practical work should be conducted with these systems to determine the differences in accuracy between them and broadband systems, particularly noting the high bandwidths of some of the LED based systems which could introduce errors into the recovery of chromophore concentrations.

### 4.4. Systems combining NIRS and DCS

There is a trend in NIRS towards systems that combine NIRS and diffuse correlation spectroscopy (DCS), enabling simultaneous blood flow and chromophore concentration measurements. This is especially interesting for studies that measure oxCCO as the blood flow measurement can be used to make a more accurate assessment of the underlying physiology during the measurement period.

• NneMo [47–49] is a combined bNIRS and DCS system. A multiplexing shutter cycles between the two systems in 3s intervals, with each achieving an acquisition time of 2.5s. The bNIRS system uses a halogen light source to supply light between 680-845nm to the head, and three detector bundles to funnel the light to a spectrometer. The DCS system comprises a long coherence laser and four fibers directing output to single photon counting modules.
• The hNIRS Ryerson system [50] uses two separate spectrometers - one to determine the concentrations of light from a 1cm channel and the other from a 3cm channel. Each has a sampling rate of 2Hz and is set to 650-1100nm. This system was used in conjunction with the Equanox 7600, which uses four wavelengths to determine an index of blood flow via DCS.
• The hsNIRS Ontario system [51] uses a filtered halogen source and two spectrometers for light detection at two separations (1cm and 3cm). The DCS module collected reflected light from a long coherence laser using four fibers connected to a single photon counting detector.

Since 2016, there has been increasing interest in using combined NIRS-DCS systems. Future work with such systems could reveal interesting information about the relationship between physiological changes and NIRS-derived oxCCO concentration changes.

## 5. Analysis of cytochrome measurements

A total of 34 papers were found that made a measurement of cerebral oxCCO in humans using NIRS between 2016-2023. These studies have been categorized into functional neonate/infant, clinical neonate/child, functional adult and clinical adult studies. The systems, paradigms and results are summarized for each category in the following sections.

### 5.1. Spatially localized oxCCO increases observed in neonates during auditory and visual stimuli

**Fig. 6.**
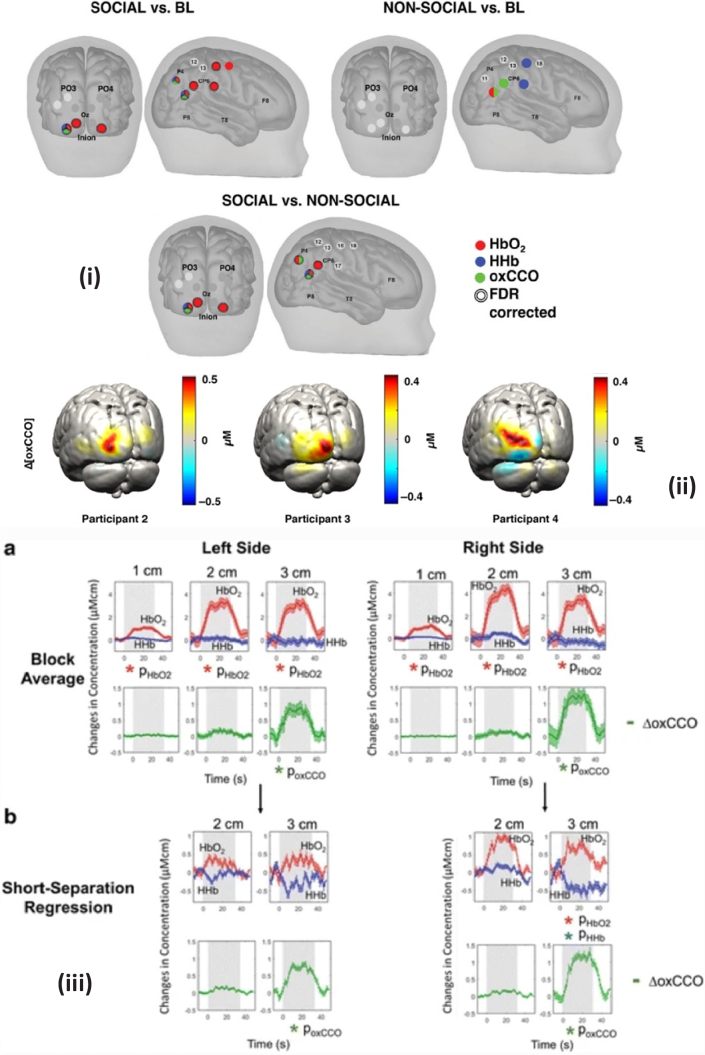
Image of notable oxCCO changes seen in studies found for this review. (i): Channels with statistically significant responses for (Top, Left) the Social condition versus baseline, (Top, Right) the Non-social condition versus baseline and (Bottom) the Social versus the Non-social condition for HbO2 (red), HHb (blue) and ΔoxCCO (green). The double line around the channel indicates statistical significance after FDR correction. [53] , (ii): Examples of reconstructed Δ[oxCCO] images on the GM surface mesh for participant 2, 3, and 4. The reconstruction for 20-s post stimulus onset is displayed. [29]. (iii): Block-average and standard error of mean for 17 subjects during functional activation for left and right sides (a) without regression (b) with short-separation regression. Stimulus period indicated by grey background. [62] .

Siddiqui et al. conducted two studies examining the response of the infant brain to visual and auditory social stimuli. The first study, published in 2017 [52], used the miniCYRIL system to obtain the changes in concentration using a single channel, and the second published in 2022 [53] used the UCL pHOS system to obtain topological images of infant brain responses. Both studies found that the presentation of the social stimuli with sound resulted in an increase in HbO and oxCCO in identical locations, and a concurrent decrease in HbR in the region of interest. In both studies, these changes in chromophore concentrations were statistically significant compared to the baseline condition of non-social stimuli. Results from the topological study are reproduced in Fig. 6(i).

It would be expected that the HbO and oxCCO concentration would increase in brain areas that were being stimulated as there is an increased oxygen demand in that area. Similarly, there would be a decrease in the concentration of HbR. However, changes in hemoglobin concentration can be influenced by extracerebral fluctuations as well as systemic changes in physiology as well as local changes in neuronal activity. As CCO is found in higher concentrations in cells within the brain (neurons and glial cells) than extracerebral tissues, changes in oxCCO are more likely to reflect changes in neuronal activity and so combining this measurement with HbO/HbR is likely to reduce the physiological noise that act as confounders [54].

### 5.2. oxCCO correlates with physiological measurements in unwell neonates and children

Seven papers were found measuring oxCCO in neonates in a clinical setting. These studies document NIRS-measured changes during spontaneous changes in systemic physiology in a variety of clinical conditions.

Mitra et al. [55] published a case study from a newborn infant with hypoxic-ischemic encephalopathy (HIE) and intractable seizures post-hypothermia treatment, using the CYRIL system. Between seizures, the oxCCO baseline progressively declined which the authors suggest results from a decrease of mitochondrial oxidative metabolism. They also reported a correlation between oxCCO and EEG voltage, as well as HbD. Another more recent study [37] observed an increase in oxCCO during seizures in a three-year old girl at the seizure focus. One hypothesis is that in some seizures, neurovascular coupling remains intact and can meet the increased oxygen demand. However when there is underlying brain damage and prolonged and frequent seizures, this mechanism can break down leading to increased oxidative stress as seen by a decline in oxCCO, causing further brain injury.

In a larger study of neonates with HIE, published by the same group, cerebral oxidative metabolism was monitored during hypothermia treatment also using the CYRIL system by Bale et al. [56]. They describe differences in cerebral oxidative metabolism depending on injury severity; in infants with spontaneous desaturations, those with more severe injury were more susceptible to oxidative stress as seen by a linear dependence of oxCCO on HbD. They also found a correlation between oxCCO and the Lac/NAA ratio derived from proton-magnetic resonance spectroscopy ([1]H MRS). In additional studies, the relationship between oxCCO and brain tissue oxygen delivery (HbD = HbO-HbR) during rewarming of neonates with HIE has shown a stronger correlation with increasing severity of brain injury [57]. Another study [58] found that pressure passive changes in cerebral metabolism and reactivity and mean arterial blood pressure (MABP), correlated with recovery from HIE at a one-year follow-up. Overall, these studies suggest that when the oxCCO signal is highly coupled to changes in systemic physiology it is indicative of more severe brain injury.

While the majority of published neonatal studies have focused on term infants with HIE, two studies by Rajaram et al. [47,49] focused on preterm and low birthweight infants. The 2020 study aimed to improve the timing of ventricular tap procedures in preterm infants with post hemorrhagic ventricular dilatation and demonstrated the feasibility of measuring cerebral oxCCO using the NneMo system. The subsequent 2022 study focused on quantifying cerebral perfusion and metabolism in low-birth-weight neonates using the same system and investigated the correlation between oxCCO, CBF, and StO_2_ using wavelet coherence analysis. They found that changes in oxCCO remained independent of CBF.

In summary, while the number of published studies is small, the data shows the utility of measuring oxCCO to provide insight into underlying pathophysiological measurements in preterm and term infants at risk of brain injury. The predictive value of oxCCO with [1]H MRS outcome data is of particular interest and warrants larger studies with longer term follow up of infants.

### 5.3. Functional studies in adults

Functional measurements of oxCCO in adults were found in 11 papers. They are summarized in sections categorized by the type of stimulation, brain area measured or processing method – visual task, cognitive attention task, Stroop task, muscle measurements and correlation analyses.

#### 5.3.1. oxCCO increases during visual stimulation

All visual stimulation studies reviewed showed a distinct increase in oxCCO in the region of the activation, contributing to the hypothesis that an increase in the oxygen demand of tissue will lead to an increase in localized cerebral metabolism for visual stimulation.

In one of the first examples of exploiting DOT to image oxCCO, reconstructed images of oxCCO, HbO and HbR were obtained during a visual stimulation study using the UCL pHOS system [29]. oxCCO was seen to increase across all participants during the visual task as shown in Fig. 6(ii). A residual analysis of a two- and three-chromophore fit confirmed the setup and paradigm was successful in reconstructing a true oxCCO signal.

oxCCO has also been seen to increase during visual tasks using other imaging systems. The CYRIL system was used to collect simultaneous NIRS and EEG data from 13 adults in a 2020 study by Pinti et al. [59] and a pipeline was presented for processing these data together to model neurovascular coupling. The ability of the 8λ UCL system to extract topological images of oxCCO was also tested using a visual activation paradigm [42]. oxCCO was observed to increase in the contralateral region, with a concurrent normal hemodynamic response observed in the hemoglobin signal.

#### 5.3.2. Changes in oxCCO during cognitive processing and attention

Cognitive attention studies characterize the response of the brain as it attempts to focus on a known relevant stimulus whilst excluding irrelevant distractions. During cognitive attention studies, cerebral oxCCO and HbO would be expected to increase in line with increasing cerebral oxygen and metabolism demands. One such study by Nosrati et al. [38] measured oxCCO in the pre-frontal cortex of 16 adult volunteers during a driving task both with and without distractions. In the distracted driving periods, they observed an increase in HbO and oxCCO and decrease in HbR, opposite to the response seen in the non-distracted driving period. The observed response during the distracted period confirmed the hypothesis that distracted driving activates the pre-frontal cortex more than non-distracted driving. The observed decrease in oxCCO during the non-distracted driving periods may be a result of the complexity of the cognitive processes associated with driving as the task stems from other more relevant areas of the brain. As the driving task does not require active attention, an increase in oxCCO and HbO may not be seen as there is not an elevated energy demand in the pre-frontal cortex.

Bruckmaier et al. [60] measured cerebral oxCCO with the CYRIL system during high and low-load visual tasks in the visual cortex of 16 adults in their 2020 study. Participants were engaged in a visual search task, and in the high visual load trials they were shown a task-irrelevant, peripheral checkerboard. Results revealed an increase in oxCCO during the high load trials, which the authors claim adds evidence to the cause of inattentional blindness.

A proof-of-concept study demonstrated the ability of a custom bNIRS system (SCISSCO) to quantify changes in HbO, HbR and oxCCO [41]. The oxCCO spectra used to quantify concentration changes was measured as part of the study by measuring the oxidized and reduced state of a prepared bovine heart. A preliminary blood pressure study was performed with the system, alongside a breath holding study and cognitive attention study. During the cognitive attention task, they observed a paradoxical decrease in oxCCO and increase in HbO when results were averaged across 25 participants. However, a disadvantage of the SCISSCO system was its long acquisition time of 17s. The authors also removed non-increasing HbO as outliers which could have confounded the results. These results from this study differ from the findings of Nosrati and Bruckmaier by observing a decrease in oxCCO during periods of increased mental demand.

The observed changes in oxCCO are inconsistent across different attention studies, with neither an increase or decrease in the signal accepted as standard. Despite some observations of an increase in oxCCO, some past studies have also observed decreases in the signal [11]. The nature of the mechanisms supplying elevated energy demands in the pre-frontal cortex are not properly understood, and further studies in this area could aid in rectifying this.

#### 5.3.3. Localized increases in oxCCO during active tasks

Stroop tasks involve identifying the actual color of a color word when there is a mismatch between the two, with the delay in reaction time known as the Stroop effect. Using the CYRIL system a significant increase in HbO and oxCCO and corresponding decrease in HbR was observed during the Stroop task [61]. By using several different channel separations it was possible to measure the response at different cortical depths. This was the first observation of an oxCCO response that is distinct from the hemoglobin signals simultaneously measured over several brain locations. The CYRIL system was utilized in this context again during a Stroop and anagram solving task [62]. An improvement in the hemodynamic signals was achieved by applying short-channel regression but this was not observed in the oxCCO signals; a significant increase in oxCCO was observed at longer distances (3cm) but not short separations (1 and 2cm) during the tasks. These results showed a very compelling, brain-specific oxCCO response visualized clearly alongside the hemoglobin response. Their results are reproduced in Fig. 6(iii).

Another study measured the pre-frontal cortex of 5 participants using the 4λ South Korea system while they solved a Sudoku puzzle [46]. Results with Cohen’s d-values greater than 0.8 and correlation coefficient greater than 0.3 were included in the analysis, with results that did not meet these criteria discarded. Consequently, the authors report observing a significant increase in HbO and oxCCO and decrease in HbR, and reason that the strong correlation between oxCCO and HbD verifies the observation of true brain activation.

### 5.4. Muscle measurements of oxCCO

The MAESTROS (TD-UCL) system was used to monitor chromophore changes during forearm ischemia achieved via rapid venous and arterial occlusion [40]. Chromophore concentration was reported as an average across six participants both 2.5 and 5 minutes after the occlusion started. At 2.5 minutes, the oxCCO concentration had decreased to its minimum value. At five minutes, the change in oxCCO concentration remained at a decrease and was largely independent of the hemoglobin response. They reported that the small observed change in oxCCO concentration indicates the absence of crosstalk as it does not mirror the hemoglobin signals. oxCCO has also been seen to remain constant despite a decrease in HbO during forearm occlusion using the SCISSCO system [41]. oxCCO and HbO were also observed to decrease during a finger occlusion in a single subject using the 4λ South Korea system [46].

A measurement of oxCCO was made in the leg muscles of marathon runners during an arterial occlusion using miniCYRIL [63]. They measured the runners using the miniCYRIL system both 16 weeks before the London marathon and three weeks after. The findings showed that the rate of concentration change of oxCCO increased with the level of training undertaken despite there being no change in cardio-respiratory fitness (measured as peakVO_2_). During the occlusion, oxCCO concentration decreased for both conditions, however the group median for rate of change of oxCCO was more negative after marathon training. These findings agree with the study using TD-UCL to measure forearm ischemia, however this is the only occlusion analyzed which performed the arterial occlusion on a muscle in the leg.

Participants undergoing arterial occlusion and venous stasis measured using the NIRO-300 showed increasing oxCCO responses which were independent of the HbO signal [44]. A cuff occlusion and motor task paradigm were used to assess the sensitivity of the 4λ Zurich system to changes in oxCCO [45]. The results of the cuff occlusion reveal a small increase in oxCCO, whereas HbO and HbR decrease and increase respectively by over ten times the amount. The results of the motor task show a distinct hemodynamic response and increasing oxCCO at both short and long channel separations. The graph displaying the result of short channel regression shows a similar expected hemodynamic response but does not include oxCCO.

The results across these studies are variable and are highly dependent on the positioning of the NIRS optodes, the body composition of participants, and the wavelengths used. It is notable that the study using the NIRO-300 discrete-wavelength system observed increasing oxCCO concentrations and the studies utilizing broadband systems observed oxCCO to decrease. Further studies in this area are required to validate the findings.

### 5.5. Correlation analyses

A frequency domain analysis was employed to investigate the coherence of infra-slow oscillation (ISO) signals in the brain in an exploratory study in 2022 [64]. Changes in HbO and oxCCO were resolved in different neural frequency bands over a 5-week (once per week) period in healthy volunteers with the bbNIRS Texas system, aiming to establish new biomarkers. The study identified 8 potential key biomarkers (correlations between several parameters involving oxCCO) but acknowledged the impact of using a single channel system with a low sampling frequency on the accuracy of their findings.

### 5.6. Adult clinical

A total of 9 studies were found which measured oxCCO in adults in a clinical setting. The following sections cover papers where patients are undergoing paradigms designed to produce changes in systemic physiology and cardiopulmonary bypass or suffering from brain injuries.

#### 5.6.1. Paradigms designed to produce changes in systemic physiology

Changes in tissue oxygenation tend to yield a concentration change in cerebral oxCCO measurable by NIRS. Hypo- and hypercapnia simulate sudden large changes in oxygenation particularly in patients requiring mechanical ventilation such as during surgery, requiring intensive care and/or following brain injury. Hypocapnia can be induced by hyperventilation which lowers the CO_2_ level in the blood resulting in vasoconstriction and hence decreases the oxygen supply of tissues. During hypocapnia, oxCCO would be expected to decrease as there is less available oxygen in cerebral tissue. Hypercapnia produces the opposite effect by causing CO_2_ to build up in tissues resulting in vasodilatation, which in turn causes red blood cells to offload oxygen to tissue, leading to an observable localized increase in cerebral tissue oxygenation and hence oxCCO. Hypercapnia effects can be induced in healthy subjects via breath holding. A paper by Holper et al. [65] aimed to explore the test-retest reliability of the oxCCO measurement using these physiological effects. Their series of hypo- and hypercapnia challenges yielded an oxCCO response over 10 participants as measured using the NIRO-300 system, and a similar Bland-Altman plot measured intraclass correlation coefficient (ICC) to HbO/HbR. The authors note that using a four-wavelength NIRS system could result in low wavelength resolution and crosstalk. A similar study [42] using the 8wav UCL system showed increased HbO and oxCCO and decreased HbR during hyperoxia.

Thirty patients with clinical depression were monitored using the NIRO-300 system and compared to healthy control subjects and observed a decrease in oxCCO during hypocapnia and an increase during hypercapnia [43]. This response is consistent with that observed in a similar 1999 study with the NIRO-500 [66]. Clinically depressed patients were however found to have lower oxCCO activity as the percentage change in this signal was around four times lower than in healthy controls. The authors hypothesize that mitochondrial abnormalities may contribute to brain disorders [67].

A measurement over the pre-frontal cortex was made with the Toronto hNIRS system in adults undergoing a BH paradigm where oxCCO was seen to increase in all three of their channel separations (10, 30 & 40mm) [68]. The largest change was seen in the 30mm-10mm channel, and a smaller change in the 40mm-10mm channel. An evaluation of the contributions from the scalp on NIRS measurements was made using the Ontario hsNIRS system [51]. Their focus was on monitoring metabolism during cardiac surgery; thus, they designed their experiments to include two paradigms – a carotid compression and hypercapnia. The carotid compression paradigm mimics the arterial occlusion performed during cardiac surgery, and hypercapnia was chosen for its vasodilatory effects in the brain. During carotid compression, oxCCO decreased in line with StO_2_, and during hypercapnia, oxCCO concentration increased. Responses were still significant upon application of a short-channel regression. Note that different wavelength ranges were used to resolve HbO & HbR (680-850nm) than oxCCO (770-906nm).

The oxCCO signal has been measured during a high + Gz acceleration using the miniCYRIL system [69]. Authors describe observing both an increase and no change in the oxCCO signal in two different participants during the centrifuge-induced acceleration and hypothesize the increase could be explained by a lack of sufficient delivery of glucose substrate to the mitochondrial respiratory chain caused by acceleration-induced ischemia, rather than an absence of oxygen.

For these paradigms, there is a consensus that hypocapnia causes a decrease in measured oxCCO, and hypercapnia an increase, agreeing with the current theory of the oxygenation and metabolism relationship. Some older studies have seen identical responses with bNIRS systems at a fixed depth [70,71] and with discrete wavelength systems [66].

#### 5.6.2. Measuring oxCCO in brain injury during hyperoxia

The response of the brain to stimuli after being subjected to injury is of great interest to clinicians as a greater understanding of the relationship between observable parameters and outcome could help inform time-critical treatment and care. One study aimed to address whether rectifying localized cerebral oxygen deficiencies had any effect on the oxygenation state of mitochondria [35]. The UCL pHOS system was used in traumatic brain injury (TBI) patients to measure changes in concentration of oxCCO, transcranial Doppler ultrasound measured cerebral blood flow velocity and cerebral microdialysis derived lactate-pyruvate ratio (LPR), brain tissue pO_2_ (pbrO_2_), and tissue oxygenation index. The patients were presented with a normobaric hyperoxia challenge 24-72 hours post-injury. The measured oxCCO increased as inspired oxygen increased from 30-100% and remained elevated while other measured parameters returned to baseline. The authors comment that while it is clinically sensible to avoid mitochondrial hypoxia, no long-term beneficial effects of hyperoxia have been identified due to lack of further assessments after this period.

#### 5.6.3. oxCCO decreases during cardiopulmonary bypass

During transcatheter aortic valve insertion, rapid ventricular pacing is required to temporarily induce sudden hypotension and hypoperfusion that mimic cardiac arrest. A rapid decrease in oxCCO and HbO and increase in HbR were observed in one study during rapid ventricular pacing [50]. Additionally, they found that while tissue oxygen saturation changes in the cerebral tissue were lower than those in the scalp during rapid ventricular pacing, oxCCO changes in the cerebral tissue were larger than those at the scalp. They thus concluded that oxCCO is a more specific biomarker of cerebral status than StO_2_. Simultaneous CBF and oxCCO measurements were made using the NNeMo system in patients receiving a cardiopulmonary bypass (CPB) procedure [48]. Across five datasets, an increase in CBF and decrease in oxCCO was observed during the bypass surgery, and changes in the CBF index of over 70% resulted in large decreases in oxCCO. The authors surmise that such measurement tools could be used to inform changes to the CPB flow rate during surgery and decrease postoperative neurological injuries.

#### 5.6.4. Clinical summary

Studies consistently observed decreased oxCCO concentrations during hypocapnia and increased oxCCO during hypercapnia. Patients with clinical depression showed lower oxCCO activity compared to healthy controls. Monitoring oxCCO during a breath holding paradigm and carotid compression/hypercapnia experiments also yielded significant changes. Additionally, oxCCO signals were measured during high + Gz acceleration, showing varied responses.

In brain injury studies, normobaric hyperoxia challenges were conducted in traumatic brain injury patients. oxCCO concentration increased with higher inspired oxygen levels. However, the long-term effects of hyperoxia require further investigation.

Rapid ventricular pacing during cardiopulmonary bypass caused decreased oxCCO. Simultaneous measurements of cerebral blood flow and oxCCO during bypass surgery revealed increased blood flow and decreased oxCCO, suggesting potential applications for monitoring and reducing postoperative neurological injuries.

## 6. Discussion and future directions

Interest in measuring oxCCO as a biomarker of metabolism has persevered in recent years. Since 2016, 34 studies have been conducted focusing on oxCCO measurements and their applications in various fields, including neuroscience, neurology, neonatology, and cognitive psychology. Several new groups have emerged with new systems for measuring oxCCO and are demonstrating their utility in human experiments. Some systems have attempted to obtain 3D functional images of oxCCO by positioning optodes at multiple source-detector separations [29,53] however this is made difficult by the quantity of fiber-optic cabling required and the current size limit of spectrometers.

Studies measuring oxCCO in neonates have found that, in a healthy brain, oxCCO increases in response to social stimuli [53]. In term infants with brain injury, changes in oxCCO was found to correlate with Lac/NAA ratio [72]. In healthy adults, increasing oxCCO was associated with increased mental processing demand in several functional paradigms [29,38,60–62]. oxCCO was also found to correlate with systemic physiology in adults undergoing various clinical challenges [35,43,48,50]. Interestingly, measurements of oxCCO are made mostly in the adult or neonate population, with a few studies conducted in infants, children or the very elderly. Functional NIRS is widely used in infants and children [73] and with more systems capable of measuring oxCCO one would anticipate its application will become more widespread in this population. Likewise, given the global health problem associated with neurodegeneration, measurement of oxCCO offers potential for a way to assess brain health in an inexpensive, non-invasive way [18].

Some paradigms show a variable oxCCO response in both social and clinical settings, rendering clinicians hesitant to adopt the technique for monitoring on a case-by-case basis. Efforts should be made to compare the oxCCO signal with other clinical markers such as fluctuations in systemic physiology, EEG or [1]H MRS derived signals and with meaningful long term outcome measures. To improve clinical utility larger multi-centre clinical trials are required.

There has been an emergence of numerous new NIRS systems to measure oxCCO [41,64] which have been trialed in animal and human studies. Several devices have combined NIRS with other techniques such as DCS, which provides additional data in terms of assessment of perfusion as well as oxygenation and metabolism. There is a shift towards systems capable of obtaining images of activity with multiple multi-distance channels. These advancements have enabled more precise and localized measurements of oxCCO. A portable, high-density multi-wavelength system capable of providing whole-head measurements would represent a new standard for imaging of oxCCO. Several groups are attempting to miniaturize time-domain technology to obtain absolute concentration data [40,74].

Translating small exploratory studies into a widely utilized clinical tool is challenging. Consensus and uniformity of measurements between different systems and manufacturers is required. Studies across large patient groups will be required and for a measurement to be clinically valuable it needs to be related to a meaningful outcome - and this can be difficult to demonstrate. Efforts in multi-center clinical trials have been demonstrated with other NIRS techniques [75].

The expanding number of research groups and measurement systems investigating oxCCO, coupled with the growing interest in oxCCO as a biomarker, presents a promising outlook. However, the lack of standardized systems and measurements hampers the widespread approval and adoption of this technique. Establishing a consensus on suitable measurement wavelengths, initial testing protocols, and standardization methods for each system is imperative. While efforts have been made to determine optimal measurement wavelengths [32,76] and SNR [77], further investigations are needed to understand the combined influence of wavelength choice, system design, layout, and processing methods on the acquired oxCCO signal. To serve as an initial proof-of-concept test, measurements of oxCCO should ideally be performed using reproducible dynamic liquid phantoms, as demonstrated in recent studies [78] and currently under investigation by our research group in Cambridge. Utilizing phantoms allows for precise control of measurement conditions and assessment of the system's ability to accurately quantify oxCCO. During phantom measurements, direct comparisons between different systems, particularly when introducing an upgraded system for oxCCO measurement, are recommended. Research groups employing multiple systems should embrace this strategy to directly evaluate their efficacy and performance.

Data processing methods in oxCCO measurements are undergoing continuous development to enhance result accuracy and spatial localization [79]. However, there is considerable variation in filtering and averaging techniques employed among studies and research groups, hindering the accurate evaluation of result quality. To address this, numerous open-source processing pipelines tailored for NIRS data have been shared in recent years [80,81]. It is crucial to establish inclusion and exclusion criteria for result selection prior to data collection, avoiding the exclusion of trials that contradict the hypothesis as outliers during group analyses.

Rigorous assessment of measurement errors is essential. The effect of the LED bandwidth on results has not been discussed in any papers using LED-based systems, despite reporting the FWHM values. A convolution method to obtain a weighted value of the extinction coefficients based on the LED bandwidths could be performed to minimize this source of error. It is very difficult to obtain information on the errors of the extinction coefficient values used in calculations, and moreover there is lack of consensus on which extinction spectra to use for each chromophore due to a lack of standard open-source databases. These are problem which should be addressed by the NIRS community. Additionally, sensitivity analyses should be conducted to evaluate the precise correspondence between optode locations and cerebral landmarks [82].

Studies with a biased participant selection can hinder the reproducibility of results and draw conclusions that are not generalizable. Differentiating between data quality associated with different skin and hair colors allows for a greater understanding of the limitations of certain systems and paradigms to obtain cerebral oxCCO signals. Studies should aim to describe their procedures for eliminating these sorts of biases.

There is an effort to work towards standardization in the NIRS field [83] and integrating specific standards for best-practice measurement of oxCCO is necessary as well. In general, validating systems first on dynamic phantoms allows conditions to be controlled and characterization of the system response more comprehensive. A study providing a proof of concept for a system to obtain oxCCO signals in a single subject does provide valuable insights. However, system validation with phantom measurements should first be performed, allowing studies in larger populations to take precedence, permitting group analyses to afford a greater impact on the understanding of oxCCO responses.

## 7. Conclusion

In this review, we described the latest technology, methods and analyses employed to measure oxCCO in humans using NIRS since 2016. We found a total of 34 published papers on oxCCO NIRS measurements in humans from 9 research groups using 15 different NIRS systems. Two thirds of the studies focused on adults and most of the rest were in neonates; it is interesting that there is a paucity of studies in infants, children and the very elderly as this could be a fruitful area of future research. The most notable area of development has been towards wearable systems capable of imaging oxCCO, which could enable functional maps of metabolism to be obtained during a variety of real-world tasks. While the recently published studies have shown that oxCCO correlates with several physiological parameters and hemodynamics in both neonate and adult populations, the variability in oxCCO response in different settings and the lack of standardization limits the potential of oxCCO as a widespread clinical biomarker. Hence there is a need for future studies to actively address these issues in order for Jöbsis’ initial vision for the biomarker to be realized.

## Data Availability

Data underlying the results presented in this paper are not publicly available at this time but may be obtained from the authors upon reasonable request.
